# Potential of Nanocellulose as a Dietary Fiber Isolated from Brewer’s Spent Grain

**DOI:** 10.3390/polym15173613

**Published:** 2023-08-31

**Authors:** Abraham Azael Morales-Juárez, Luis Daniel Terrazas Armendáriz, Juan Manuel Alcocer-González, Leonardo Chávez-Guerrero

**Affiliations:** 1 Mechanical and Electrical Engineering School, Universidad Autónoma de Nuevo León, Pedro de Alba s/n, San Nicolás de los Garza, San Nicolas de los Garza C.P. 66455, Nuevo León, Mexico; azael05_1591@hotmail.com; 2 Biological Sciences School, Universidad Autónoma de Nuevo León, Pedro de Alba s/n, San Nicolás de los Garza C.P. 66455, Nuevo León, Mexico; luis.terrazasarmn@uanl.edu.mx (L.D.T.A.); juan.alcocerg@uanl.mx (J.M.A.-G.)

**Keywords:** cellulose nanosheets, dietetic fiber, weight control, beer by-products

## Abstract

Steady growth in beer production is increasing the number of by-products named brewers’ spent grain. Such by-products are a source of several components, where cellulose is usually present in high amounts. The aim of this study was to develop a protocol to obtain a mix of cellulose microfibers with an average diameter of 8–12 µm and cellulose nanoplatelets with an average thickness of 100 nm, which has several applications in the food industry. The process comprised one alkaline treatment followed by acid hydrolysis, giving a new mix of micro and nanocellulose. This mix was characterized by Fourier transform infrared spectroscopy, scanning electron microscopy, and laser scanning microscopy corroborating the presence and measurements of the cellulose nanostructure, showing an aspect ratio of up to 500. Finally, we demonstrated that the administration of this new type of nanocellulose allowed us to control the weight of mice (feed intake), showing a significant percentage of weight reduction (4.96%) after 15 days compared with their initial weight, indicating the possibility of using this material as a dietary fiber.

## 1. Introduction

Alternatives to fulfill the energy and food demands are continuously sought by researchers trying to achieve high manufacturing standards for various daily-use products [1]. Most industries deplete resources at a very high rate, thereby critically modifying and contaminating ecosystems, which requires immediate action. One strategy to address this problem is through the circular economy by recycling and reusing byproducts [2], which have economic and environmental benefits. The brewing industry is responsible for huge water consumption along with a large production of solid and liquid byproducts [3]. The solid by-products are yeast and malted barley bagasse or brewer’s spent grain (BSG); these materials represent a significant amount of by-products with a BSG generation of 20 kg/hl [3,4]. In 2017, the global amount of produced beer was 1.95 billion hectoliters, with Mexico as the largest exporter [1]. Such by-products (BSG) have various applications, mainly used as food for livestock [5,6]; they are also used for food supplements, biogas generation, concentrated protein production [5,6,7], biofuels [4], particleboard [8], among others [9]. The large amount of BSG is generated when the wort is separated from the spent grain, which is a major reason it has to be reused or processed, following a circular economy, bearing in mind that 80% of BSG content is liquid, which is a huge limitation due to transport costs (cattle food supplement). During maceration, the goal is to extract starch [4,10]; therefore, most components of the grain remain, e.g., proteins, amino acids, minerals, and husks—comprising polysaccharides and lignin [6,8,11,12,13,14]. Among polysaccharides, cellulose (16–26%) and hemicellulose (14–35%) are the main components, along with lignin (7–28%) and inorganic residues (8–15%). The great variation of the components is due to the growth conditions of the barley, where many factors can be mentioned as the type of grain, region of precedence along with the weather conditions while growing.

Cellulose is a biopolymer composed of anhydroglucose units linked by a β-1–4 glycosidic bond, whose properties depend on the source and treatment applied to their isolation [15], and can be used in applications such as dietetic fiber [16,17], bioplastics, or transparent films [2,3,5,6]. Some advantages of cellulose and nanocellulose are their abundance in nature, low density, biocompatibility, nontoxicity, biodegradability, low coefficient of thermal expansion, and high Young’s modulus [15].

Several methods have been reported to isolate nanostructures, e.g., cryo-grinding, ultrafine grinding, high-pressure homogenization, chemical treatments [18], ultrasound, enzymatic treatment, and oxidation by TEMPO [19,20]. These methods allow the isolation and production of cellulose with different shapes and sizes, such as microfibers, nanowhiskers, nanofibrils (CNF) [15,20], and nanoplatelets (2D) [18]. However, most routes are highly polluting and time-consuming, with high energy demand, causing their production to be slightly viable due to the use of high volumes of very sophisticated equipment or slow processes. In this study, the BSG is used to isolate cellulose and nanocellulose through alkaline pretreatment, followed by one-step acid hydrolysis. In addition, we show a prelaminar work on the effect of this material as a dietary fiber, indicating a weight reduction when tested on mice over time.

## 2. Materials and Methods

### 2.1. Materials

The waste from the brewing process (BSG) was provided by a local brewery, using malt without any other grain. The concentrated sulfuric acid (98%), hydrogen peroxide (30%), and sodium hydroxide (pellets) were reagent grade and used as received.

### 2.2. BSG Pretreatment

The BSG was washed with deionized water to remove residual sugars. The sample was dried at 50 °C for 24 h losing up to 80% of its initial weight. A total of 100 g of sample was placed in a high-speed 28,000 rpm electric mill for 4 min, the grinding process was repeated 5 times; the obtained powder was separated with an All-American sieve (#150) to obtain particles smaller than 100 µm. The sieved raw sample is called BSG-Control.

### 2.3. Alkaline Treatment

A conical flask was used to dissolve 3 g of NaOH into 100 mL of H_2_O_2_ at room temperature. After 25 min of stirring, 10 g of BSG–Control was added to the solution with constant stirring (700 rpm) until the powder was completely dispersed. This process must be carried out in a glass container with at least 20 times (2 L) the volume used in the reaction (100 mL) due to the production of bubbles, a consequence of the gas release due to the interaction between sodium hydroxide and hydrogen peroxide [21]. The mix is kept at 65 °C for 60 min under constant stirring (700 rpm). Then, the sample is centrifuged at 6500 rpm for 25 min at 4 °C. The supernatant was properly disposed of and the sediment was recovered and returned to the glass bottle with 200 mL of deionized water. Then, the mix was neutralized with a sulfuric acid solution to reach a pH of 6 and again centrifuged, repeating these steps one more time. This sample was named BSG-Alkali.

### 2.4. Acid Hydrolysis

A solution with 5 mL of H_2_SO_4_ and 45 mL of H_2_O_2_ was prepared and kept at room temperature with constant stirring. The sediment recollected from the alkaline pretreatment was added to the solution in an autoclavable glass bottle (1 L) and stirred for 10 min. The bottle was introduced in an autoclave and kept at 110 °C for 45 min. Then, once the autoclave reached 70 °C the bottle was extracted and kept under constant stirring for 30 min. The sample was centrifuged at 6500 rpm for 25 min at 4 °C, the supernatant was properly disposed and the sediment was recovered and returned to the glass bottle with 200 mL of deionized water. The sample is neutralized to a pH of 6 using sodium hydroxide pellets. This step was repeated two times until the sample is free of salts. This sample was named BSG-Acid.

### 2.5. Laser Scanning Microscope (LSM) 

A drop of the nanocellulose suspension (BSG-acid) was deposited on a silicon wafer and then dried at 40 °C for 5 h. A laser scanning microscope (Confocal) was used to obtain height profiles with a ZEISS LSM 700 (ZEISS, Jena, Germany), mainly to study the topography of the samples with a laser emitting at 405 nm, using 150 layers and 512 × 512 pixels to build each image. All images were acquired with the 20× or 100× objectives. 

### 2.6. Scanning Electron Microscope (SEM)

A drop of the nanocellulose suspension (BSG-acid) was deposited on a silicon wafer and then dried at 40 °C for 5 h. Then, the sample was sputter-coated with gold in a Cressington Coater 108 Auto for 10 s using 40 A under an argon atmosphere. The morphology was studied using an FEI NOVA NANOSEM 200 (Hillsboro, OR, USA) with an accelerating voltage of 3–5 kV and a working distance of 5 mm. 

### 2.7. In Vivo Studies 

All animal handling procedures were carried out in accordance with the official Mexican standard “NOM-062-ZOO-1999, technical specifications for the production, care, and use of laboratory animals” and were approved by the Research Ethics Committee of Animal Welfare (CEIBA) of the Faculty of Biological Sciences of the Autonomous University of Nuevo León. Male mice of the BALB/c strain (8 weeks old) were purchased from Harlan Laboratories (Indianapolis, IN, USA) and were housed in standard micro isolator cages under controlled conditions of humidity, light and temperature. The mice were divided into 4 groups of four mice per group (*n* = 4). Each mouse was anesthetized with a mixture of Ketamine (87.5 mg/Kg) and Xylazine (12.5 mg/Kg). Different concentrations of Nanocellulose (NC) (360 µg, 720 µg, and 1080 µg) diluted with cream were administered intragastrically in a total volume of 120 µL on days 0 and 7. Food intake was monitored daily while the weights of mice were every 3 days until the 15th.

All data are presented as the mean ± standard error of the mean (SEM). Statistical and graphic analysis was carried out using Graph Pad Prism version 6.0 software. The normality of the data was verified, and subsequently, a post-ANOVA test was performed for the multiple comparisons of means by the Tukey test. Significance was defined as * *p* < 0.05, ** *p* < 0.01, *** *p* < 0.001 and **** *p* < 0.0001.

## 3. Results and Discussion 

### 3.1. Cellulose Isolation

**Figure 1:**
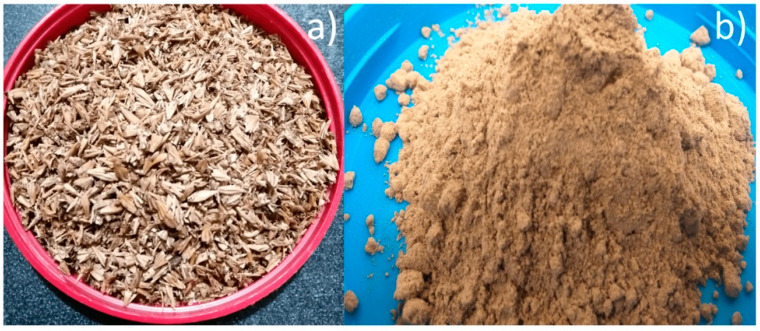
(**a**) BSG washed and dried and (**b**) BSG control after grinding and sieving.

The appearance of the raw BSG and BSG control can be observed in Figure 1a,b respectively. The grinding and sieving are mandatory pretreatments to increase the contact surface, helping to achieve the delignification in the next steps; then, better results can be obtained.

The main objective of the alkaline treatment is to remove as many as possible noncellulosic materials present in the raw material. The lignin negatively affects the appearance and properties of the final product (cellulose); therefore, it has to be removed (Table 1). Hemicellulose also affects the mechanical properties of the cellulose products due to the size of the remaining fiber affecting the optical properties caused by the high opacity when thin films are produced [6].

However, it has been reported that low concentrations can improve the mechanical properties and some characteristics of manufacturing paper and transparent films [22]. After the reaction between NaOH and H_2_O_2_ in the solution, hydroxyl radicals (OH^−^) and superoxide anions (O_2_^−^) were formed, resulting in a highly oxidative solvent when interacting with the sample [23]. Under alkaline conditions, hydrogen peroxide dissociates to form hydroperoxide anions (HOO^−^), which is a strong nucleophilic agent (Equation (1)) [21].

H_2_O_2_ + OH^−^ ↔ H_2_O + HOO^−^ (1)

However, at the moment HOO^−^ is produced, it begins to react with the H_2_O_2_, producing other oxidizing agents (Equation (2)) [23].

H_2_O_2_ + HOO^−^ → HO + O_2_^−^ + H_2_O (2)

Each oxidizing agent has a different specific type of reaction; thus, the abovementioned reactions are required. During delignification, quinones are formed; therefore, the groups responsible for giving the characteristic color [24] must be eliminated.

One essential factor to consider is that the BSG had a significantly low amount of lignin (7–15%) based on some earlier reports (Table 1), whereas recent studies suggested that the amount of cellulose in the raw material was higher, ranging from 16.8% to 25.4% (Table 1). Under the alkaline treatment, a colored (amber) can be seen in the suspension due to the presence of quinones, which are molecules responsible for giving color to different materials and are applied in the industry as staining agents. However, after the washing and neutralization steps, the removal of the color was completed, resulting in a white suspension, indicating that the alkaline treatment was efficient for partially removing the compounds that give the BSG the characteristic color. This indicates that the treatment is efficient with only one bleaching, unlike other processes where several steps are necessary both in an autoclave (up to 3 times for 45 min) and a greater quantity of reagents applied [25]; also, the required time to achieve an efficient delignification reduced.

**Figure 2:**
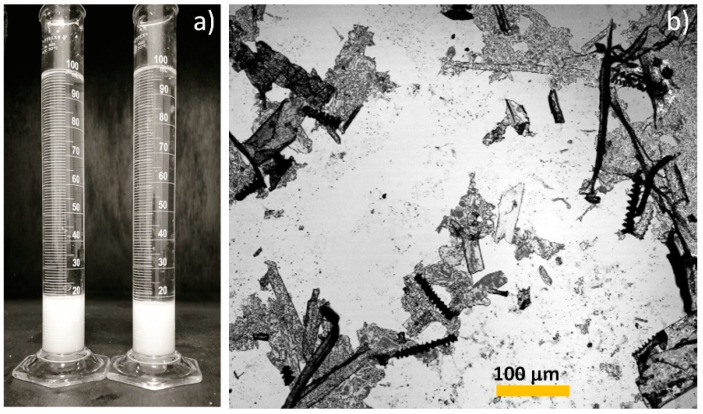
Sample after acid treatment (BSG-acid) and neutralization in (**a**) and a suspension of BSG-acid viewed under the optical microscope in (**b**).

After the acid hydrolysis, it can be seen that the color was eliminated producing a white gel (Figure 2a). Once the BSG-acid was neutralized and washed, a simple test to determine if the sample was ready was performed and verified in an optical microscope (Figure 2b). The salts were observed as well-defined crystals, where cellulose nanoplatelets (CNP) and microcellulose differed due to their characteristic shapes (fibers or platelets).

### 3.2. Fourier Transform Infrared Spectroscopy (FT-IR) Analysis

**Figure 3:**
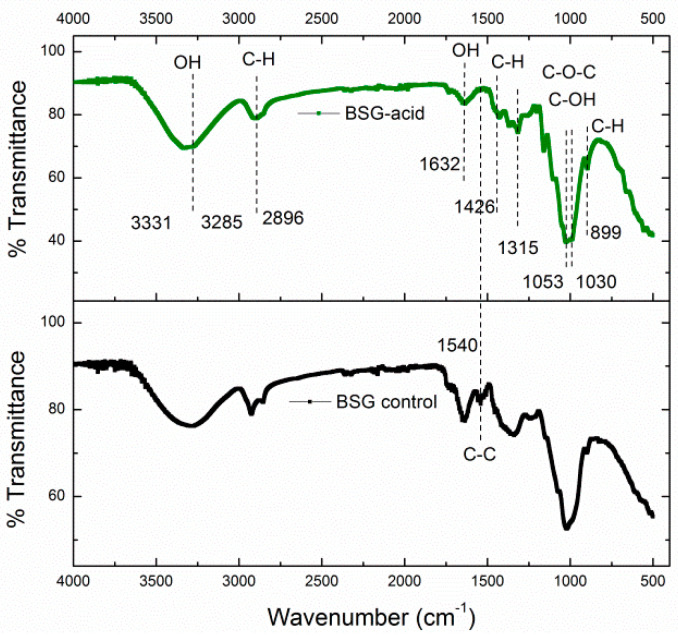
FT-IR spectrum of the samples BSG control and BSG-acid.

The FT-IR analysis indicated the presence of functional groups of the lignin (aromatic ring) and other noncellulosic elements (Figure 3). In this analysis, different bands corresponding to the cellulose were observed, demonstrating that the hydrolysis worked adequately for the removal of lignin, thereby successfully isolating nanocellulose. 

The bands found between 3200 and 3500 cm^−1^ corresponded to the OH^−^ groups in the cellulose [25,26]. An intense band was observed in 2896 cm^−1^, attributable to the stretching vibrations of the C–H bonds. The bands located between 1800 and 1000 cm^−1^ agreed with previous reports and were due to the movement and bending of anhydroglucopyranose units. The band found at 1632 cm^−1^ corresponded to the absorbed water, partially provoked by the acid hydrolysis. The band at 899 cm^−1^ was associated with the typical structure of cellulose (due to β-glycosidic links to glucose) and C–H of the oscillating movement of cellulose [26]. Finally, the bands between 1500–1600 cm^−1^ shown in BSG control are related to the C=C stretch vibration in lignin, which was not observed for the isolated cellulose (BSG-acid). 

### 3.3. Morphological Characterization

**Figure 4:**
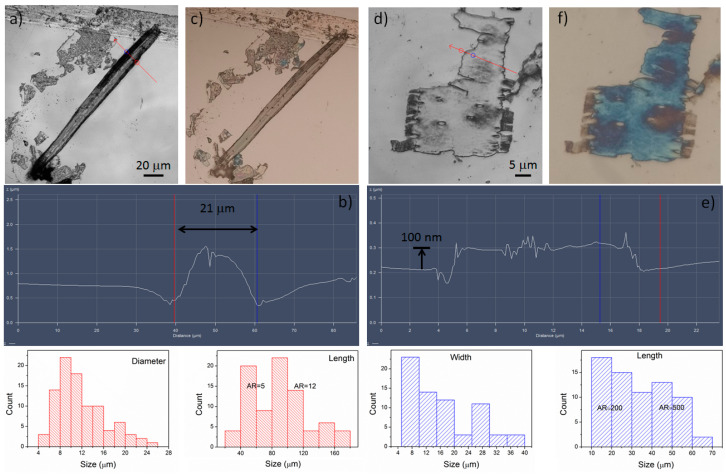
Sample of BSG-acid on a silicon wafer showing a microfiber in (**a**–**c**) and a CNP in (**d**–**f**). In both cases, the particle size distribution is presented.

Laser scanning microscopy (LSM) identified the nanoplatelets and cellulose fibers within, where several hundred microns in length were observed (Figure 4). If the hydrolyzed material containing mostly nanoplatelets is used to produce thin films, they can become brittle because there is no other polymer in the solution that serves as a bond between the nanocellulose; however, if it contains cellulose fibers, they act as a reinforcing material, improving its properties, as previously reported by different authors [27]. The dimensions of the nanoplatelets are shown in Figure 4, where the width is around 8–40 μm but the length can be up to 70 μm. Moreover, microfibers having diameters around 10 μm with lengths of hundreds of microns can be found. The detected fibers varied in lengths of 21–100 μm and diameters of 4–26 μm, which might be due to the low concentration of acid used. 

The nanoplatelets were mostly less than 70 μm in length, and the shortest side was less than 40 μm in width. The thickness of the samples (blue ≈ 100 nm) was verified by the deviation of the visible light spectrum of the sample under the illumination of the confocal microscope (Figure 4f). Then, considering a constant thickness of 100 nm, and a length of 50 µm, the aspect ratio (AR = length/thickness) could be up to 500, which is higher than that of the fiber (AR = 5–16). 

**Figure 5:**
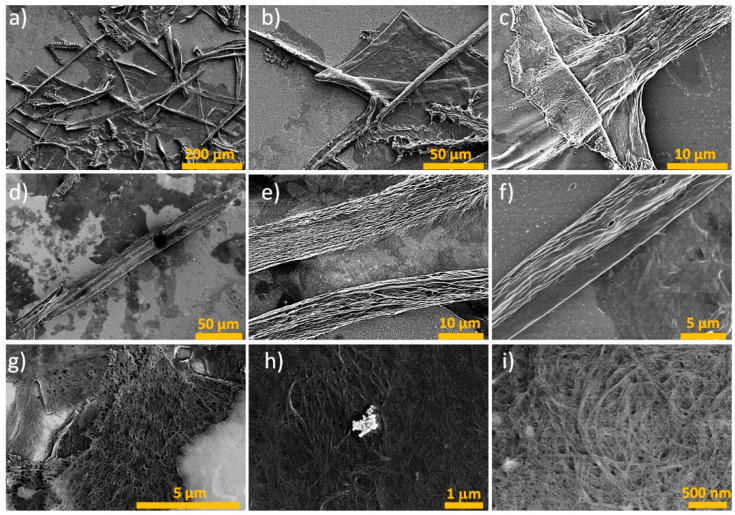
Micrograph obtained by SEM of the BSG-acid in (**a**–**c**) and individual microfibers (**d**–**f**), whereas cellulose nanoplatelets and their morphology can be observed in (**g**–**i**).

The scanning electron microscopy (SEM) analysis revealed interesting features for the nanoplatelets (Figure 5), which comprised entangled nanofibers (Figure 5g–i) that are not independent but embedded in a matrix. Here, they were exposed enough at the surface to distinguish them; these nanofibers had diameters below 30 nm. The presence of this type of morphology was due to the low concentration of the acid used. However, little is known about the biocompatibility properties of this type of nanocellulose (2D); therefore, it is necessary to conduct more in-depth studies to define its properties. In addition, the microfibers were minutely observed (Figure 5d–f), corroborating the results obtained using LSM.

The structure of the nanoplatelets has already been reported by Chávez-Guerrero et al. [28,29], where a similar methodology was used to obtain those structures, using agave as a source of cellulose. The differences in the methodologies are mainly based on the amount of lignin (and color), in the leaves of the agave (white), the lignin concentration was very low, around 9.8%, whereas the BSG (brown) varied, from 7% to 27%; with the increase in lignin, the presence of hemicelluloses also increased, causing the alkaline treatment and hydrolysis to have a higher concentration of chemicals; however, the presence of microfibers was not reported in those articles.

Despite using a high concentration of chemicals, the amount of nanocellulose is still lower than those of recently reported works in the extraction of CNF [11,23,30,31]. However, it is necessary to increase the efficiency of the process due to the presence of microfibers, which are difficult to separate into nanofibers due to the lignin that keeps them together.

### 3.4. Mouse Dietary Nanocellulose

**Figure 6:**
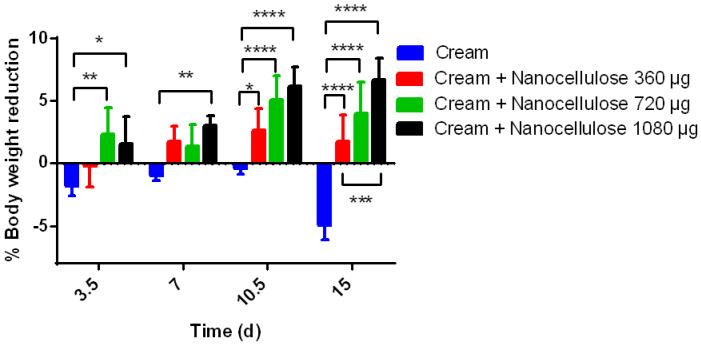
Effect of nanocellulose (CNP/microfibers) on the decrease in weight of BALB/c mice. A “positive” weight decrease percentage represents weight loss, whereas a “negative” weight decrease percentage represents weight gain. Data represent the mean ± standard error of the mean of four repetitions. Significant differences are shown (* *p* < 0.05, ** *p* < 0.01, *** *p* < 0.001 and **** *p* < 0.0001).

The group of mice that were administered cream as a diet significantly increased their weight on day 15 (% 4.9 *p* ≤ 0.0001) compared with their initial weight, whereas the groups where cream was administered plus 360 µg of BSG-acid (% 1.73 *p* ≤ 0.0001), 720 µg of BSG-acid (% 3.9 *p* ≤ 0.0001) and 1080 µg of BSG-acid (% 4.96 *p* ≤ 0.0001) showed a highly significant percentage of weight reduction compared with their initial weight and with the group that only the cream was administered (Figure 6). Notably, there was a significant difference in the percentage of weight reduction in all BSG cellulose groups (360, 720, and 1080 µg) and the group that was administered cream from day three. Some studies have obtained a significant reduction in weight, such as the case of Raza et al. [32], who reported a 60% reduction in weight at the end of 12 weeks in C57bl6 mice, where they administered a combination of cellulose and a fraction rich in lignin from BSG; they show a pattern of weight reduction in mice similar to our results.

**Figure 7:**
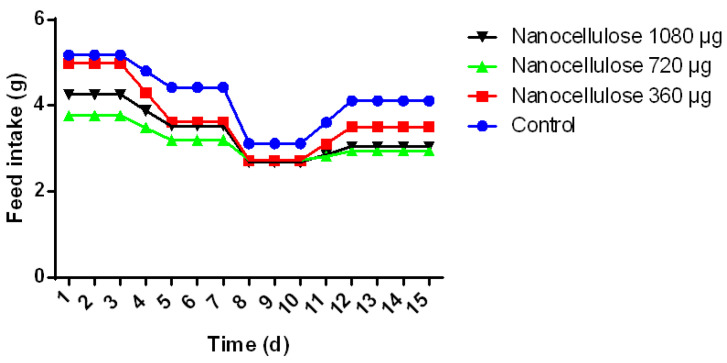
Effect of nanocellulose (CNP/microfibers) on food intake in BALB/c mice. Data are expressed as mean ± standard error of the mean of four repetitions.

In addition, food intake was monitored daily; a correlation was observed between the decrease in food consumption and the percentage of decrease in weight in the groups where 360 µg of BSG-acid (1.44 g), 720 µg of BSG-acid (0.82 g), 1080 µg of BSG-acid (1.21), and cream (1.07g) were administered (Figure 7). Cellulose and lignin residues have been shown to generate acetic acid and high concentrations of propionic and butyric acids in addition to being partially fermented in the gastrointestinal tract and producing short-chain fatty acids [33,34]. These acids stimulate the secretion of anorectic intestinal hormones, which could contribute to the effect of decreased food intake [17,32].

## 4. Conclusions

In this study, we found that the delignification process was more effective and less time-consuming than in earlier reports, as confirmed by Fourier transform infrared spectroscopy. In addition, after one alkaline and one acid treatment, the total elimination of the lignin was achieved, which could be observed in the scanning electron microscopy images. The cellulose nanoplatelets production using BSG was achieved for the first time, in a process of just two steps with a lower concentration of reagents and a significant reduction in time. We demonstrated the presence of cellulose with two types of morphology: one in the form of the usual fibers in the microscale (diameters of 4–26 μm) and another in the nanoscale in the form of platelets (thickness ≈ 100 nm). We also confirmed that the nanocellulose group (cream and 1080 µg of BSG-acid) had a highly significant percentage of weight reduction (4.96%) compared with their initial weight and other groups. We also observed that similar results could be obtained using 720 or 1080 µg of nanocellulose (BSG-based), which helped determine the minimum amount of nanocellulose to obtain the desired results. Brewer’s spent grain can be processed to obtain a mix of cellulose microfibers and can be used as dietary fiber in a mice model for weight reduction (feed intake). Further studies need to be carried out to demonstrate if cellulose microfibers have the same effect in humans.

## Figures and Tables

**Table 1:** The average amount of components usually found in the BSG.

Components	Klímek P. [8]	Berglund [11]	Buffington [12]	Mussatto [5]	Mussatto [13]	Russ [14]
Cellulose	24.5%	17%	22.2%	25.4%	16.8%	23–25%
Lignin	15.8%	21%	26.8%	11.9%	27.8%	7–8%
Hemicellulose	23.8%	29%	14.1%	n.a	28.4%	30–35%

## Data Availability

The datasets generated during and/or analysed during the current study are available from the corresponding author on reasonable request.
